# Postoperative Consequences of Fear and Anxiety in Open Heart Surgery: A Comparative Analysis of Two Patient Groups

**DOI:** 10.1097/jnr.0000000000000721

**Published:** 2026-01-09

**Authors:** Ebru DIZDAR, Aşkin KILIÇ, Semiha ALKAN KAYHAN, Dilek ÇİLİNGİR, Şeyda BABUL TURHAN

**Affiliations:** 1 Operating Theatre and Training Unit, Ahi Evren Chest, Heart and Vascular Surgery Training and Research Hospital, Trabzon, Turkey; 2 Cardiovascular Surgery Clinic, Samsun University Faculty of Medicine, Samsun, Turkey; 3 Department of Surgical Disease Nursing, Faculty of Health Science, Karadeniz Technical University, Trabzon, Turkey; 4 Department of Orthopaedic and Physical Therapy, Yavuz Selim Bone Diseases and Rehabilitation Hospital, Trabzon, Turkey

**Keywords:** anxiety, fear, pain, patient care

## Abstract

**Background:**

Patients undergoing open heart surgery may experience negative emotions such as anxiety, fear, panic, and anger in the preoperative period that may negatively affect their postoperative recovery.

**Purpose:**

This study was designed to compare the effects of preoperative fear and anxiety on postoperative pain and complications in a referral group and on a non-referral group.

**Methods:**

This descriptive study was conducted at a cardiovascular surgery hospital in Turkey from July 1, 2020, to December 31, 2020, on 96 patient participants. The referral group (*n* = 48) comprised patients who were diagnosed in another hospital and received cardiovascular surgery at our hospital, while the non-referral group (*n* = 48) comprised patients who received their angiography and cardiovascular surgery in our hospital. A Personal Information Form, Surgical Fear Questionnaire, and the Beck Anxiety Inventory were administered to all of the participants in the preoperative period. In the postoperative period, pain levels were determined using the Visual Analog Scale, and complications were recorded.

**Results:**

No statistically significant difference were found in the overall occurrence of complications between the two groups (*p* > .05). However, those in the referral group who developed lung-related complications exhibited significantly higher levels of surgical fear (*p* < .05). No statistically significant differences were observed between the groups in terms of length of stay in the intensive care unit, length of intubation, length of mobilization, length of hospitalization, pain levels, or mean anxiety and surgical fear scores (*p* > .05). A significantly positive relationship was observed between anxiety and surgical fear levels in both groups (*p* < .05).

**Conclusions/Implications for Practice:**

The results indicate neither the timing of surgical treatment decision nor preoperative fear and anxiety are associated with postoperative pain and certain complications. However, a significant relationship was detected between preoperative anxiety and surgical fear. No relationship between the way patients are taken to surgery and levels of anxiety and surgical fear or postoperative complications was found. The literature should be further expanded by conducting different studies on this subject.

## Introduction

Surgical interventions designed to cure medical conditions may result in physical and psychological complications that are potentially life-threatening. Patients face significant physical and emotional challenges during the surgical process (Sabine et al., 2022). Surgical patients are exposed to various stressors, including anxiety caused by factors such as pain, deformities, fear of postoperative dependency and death, the impact of anesthesia, job loss, and social status decline (Abate et al., 2020; Khalili et al., 2019).

Cardiac surgery can be psychologically and physically stressful on patients, causing fear and anxiety. Before surgery, surgical patients often experience physical and psychological conditions such as anxiety, uncertainty, fear, depression, worry about the outcome of the surgery, and pain (Ramesh et al., 2017; Salzmann et al., 2020). The results of prior research indicate the preoperative anxiety level in patients scheduled for cardiac surgery to be particularly high (Mudgalkar et al., 2022; Ramesh et al., 2017).

Anxiety and fear can influence individual responses to surgical interventions. The presence of fear and anxiety in patients undergoing surgery correlates with increased risk of adverse outcomes such as morbidity and mortality, delayed wound healing, prolonged hospitalization, surgical refusal, exacerbated coronary artery disease, fluid and electrolyte imbalances, infections, and increased postoperative analgesic requirements (Kashif et al., 2022; Mulugeta et al., 2018; Peker & Polat, 2021; Riecke et al., 2023; Stamenkovic et al., 2018). Preoperative anxiety not only contributes to increased pain levels but also to delayed recovery and a diminished quality of life during the postoperative phase. Prolonged negative emotions may induce cerebral artery spasms, leading to regional brain changes that intensify the perception of pain. Research indicates preoperative fear of surgery to be a key factor influencing postoperative pain (Glare et al., 2019; Riecke et al., 2023). Also, postoperative pain discourages patients from engaging in essential movements and breathing/coughing exercises, resulting in various complications (Reisli et al., 2021). Notably, preoperative anxiety contributes to a spectrum of postoperative complications, manifesting as cardiovascular disturbances (e.g., increased blood pressure and heart rate), gastrointestinal issues (e.g., nausea, vomiting, and diarrhea), and endocrine disruptions (e.g., sweating and susceptibility to infection; Wondmieneh, 2020).

After coronary artery bypass surgery, mood changes such as elevated levels of anxiety, depression, panic, and anger are commonly observed. These emotional shifts stem from psychological challenges, feelings of weakness, perceived lack of control, and diminished self-confidence. Although the negative consequences of preoperative anxiety and fear are mentioned in the literature, no study was found that specifically addresses the risks in patients scheduled for cardiac surgery. Therefore, in this study, preoperative surgical fear and anxiety, postoperative pain, and concerns about possible complications were compared between two groups, that is, patients whose coronary artery bypass surgery decision was made in a different center (patients admitted from a referral) and those whose coronary artery bypass surgery decision was made in our hospital (nonreferral).

## Methods

### Study Design

This descriptive study was conducted to determine the effect of preoperative fear and anxiety on postoperative pain and complications between referral and nonreferral.

### Sample Size and Participants

The study included two groups of patients scheduled to receive coronary artery bypass surgery. Patients referred for surgery at our hospital by nonaffiliated outpatient clinics were assigned to the referral group and those diagnosed and scheduled for this surgery at our hospital were assigned to the nonreferral group. The sample size was calculated using G*power based on a similar study in the literature (Findik & Yildizeli Topçu, 2012). The difference between the two independent means (effect size = 0.67, power = 0.9 and *df* = 96) was referenced. The study included 96 patients, with 48 in each group.

The inclusion criteria were patients at least 18 years old with no psychiatric illness diagnosis requiring potentially mood-altering treatments who were willing to participate voluntarily. The exclusion criterion was having a mental or organic disability that could impair the respondent’s ability to answer the study survey. Ethics committee approval was obtained from the Kanuni Education and Research Hospital Ethics Committee (Number: 2020/74). Written informed consent was obtained from all of the participants.

### Outcome Measures

The data were collected using a Personal Information Form, Surgical Fear Questionnaire, the “Beck Anxiety Inventory,” and the Visual Analog Scale to assess postoperative pain. Postoperative complications were monitored by the researchers.

### The Personal Information Form

The Personal Information Form used in this study, developed by the researchers after reviewing the relevant literature, is divided into two sections. The first includes 13 questions (Arslan & Emir, 2022; Khalili et al., 2019; Mulugeta et al., 2018), with seven addressing sociodemographic characteristics (e.g., age, gender, marital status, and educational status) and six addressing surgical characteristics (e.g., diagnosis, chronic disease, and previous surgery). The second section records the presence and severity of postoperative complications such as bleeding, infection, atrial fibrillation, gastrointestinal, respiratory, cardiac, neurological, and renal problems.

### The Surgical Fear Questionnaire

The Surgical Fear Questionnaire is designed to measure fear related to the short-term and long-term consequences of surgery (Theunissen et al., 2014). Validity and reliability for its use in Turkey were established by Karaman Özlü in 2018 (Bağdigen & Karaman Özlü, 2018). The Surgical Fear Questionnaire includes eight items organized into two dimensions, with items 1–4 assessing fear of short-term consequences and items 5–8 assessing fear of long-term consequences. Each item is scored on an 11-point Likert scale ranging from 0 to 10, with 0 representing *not afraid at all* and 10 representing *very afraid* (Bağdigen & Karaman Özlü, 2018; Theunissen et al., 2014). The Cronbach’s α coefficient of the total scale score was found to be .93 in the original study (Theunissen et al., 2014) and .88 in this study.

### The Beck Anxiety Inventory

The Turkish version of the 21-item Beck Anxiety Inventory (BAI), originally developed in English by Beck et al. in 1988 as a clinical anxiety measurement, was validated and assessed for reliability by Avci in 1995 (Avci, 1995; Beck et al., 1988). This scale is designed to measure the extent of symptom severity over the past week. Responses are scored as *None* (0), *Mild* (1), *Moderate* (2), and *Sever*e (3), giving the BAI a total score range of 0–63. (Avci, 1995). The Cronbach’s α coefficient for the BAI was .94 in the original study (Beck et al., 1988) and .88 in this study.

### Visual Analog Scale

The Visual Analog Scale (VAS) is the most widely employed tool for measuring pain worldwide and has high repeat-test reliability and validity. The VAS uses a 10-cm-long horizontal or vertical line along which respondents are asked to pinpoint their perceived pain level. The line in this study was graded from 1 to 10, representing the intensity of pain gradient experienced from lowest to highest (Chiarotto et al., 2019; Hawker et al., 2011).

### Data Collection

The investigator visited the patients who met the study criteria preoperatively in the cardiovascular surgery clinic and informed them about the postoperative period, taught them respiratory and cough exercises, and implemented early mobilization. In addition, the patients were briefed about the study, and a mutually agreeable time was scheduled for administering the study questionnaires in the patient’s room 1 day before the operation. The researcher read the questions from the data collection form aloud and recorded each participant’s responses. The personal information form, surgical fear scale, and anxiety scale were administered to the patients preoperatively, while pain level was measured using the VAS pain scale and complications experienced up to the time of hospital discharge were recorded postoperatively. The entire data collection process took ~20 minutes. The research was conducted between June 31, 2020, and December 31, 2020.

### Statistical Analysis

Data analysis was conducted using the IBM SPSS Statistics Version 25.0 (IBM Corp., Armonk, NY, USA). Descriptive statistics, including percentage, mean, and standard deviation, were employed. The Kolmogorov–Smirnov test assessed normal distribution conformity. Mann–Whitney *U* was used to compare the scores between groups for data that did not fit the normal distribution. χ^2^ was used for categorical variables. The relationship among groups (referral and nonreferral) and possible complications, surgical fear scores, anxiety scores, and pain scores were analyzed using the Mann–Whitney and χ^2^ tests. Correlation analysis was performed to examine the between-group relationships in terms of anxiety and surgical fear scale scores, pain score, length of stay in intensive care, duration of intubation, time to mobilization, and time to discharge. Statistical significance was set at *p* < .05.

## Results

The average age was 62 (*SD* =8.7) years and 64 (*SD* =7.3) years, respectively, in the referral and nonreferral groups. In the referral group, 75% were male, 87.5% were married, 68.8% were primary school graduates, 47.9% lived in the county, 41.7% lived with their spouses and children, and 29.2% were employed. In addition, 72.9% had chronic diseases, 60.0% had a history of diabetes mellitus, and 82.9% had a history of hypertension. Also, 58.3% reported having previously undergone surgery, and 83.3% had a history of prior hospitalization. Half (50%) of the referral group had received training before their operation, with 45.8% of these noting this training had been provided by both a physician and nurse, and 75.0% reporting the training as adequate.

In the nonreferral group, 89.6% were male, 91.7% were married, 56.3% were primary school graduates, half lived in the county, 54.2% lived with their spouses, and 10.4% were employed. In addition, 62.5% had a history of chronic disease, 63.3% had diabetes mellitus, 60.0% had hypertension, and 13.3% had chronic obstructive pulmonary disease. Also, 50% reported having previously undergone surgery, and 72.9% had a history of prior hospitalization. Over half (56.3%) of the nonreferral group had received training before their operation, with 55.6% of these noting this training had been provided by both a physician and nurse, and 70.4% reporting the training as adequate (Table 1).

**Table 1:** Descriptive Characteristics of the Referral and Nonreferral Group (*N* = 96)

Descriptive Statistics	Referral Group (*n* = 48)	Nonreferral Group (*n* = 48)	Total (*n* = 96)
	*n* (%)	*n* (%)	*n* (%)
Age (year; Mean ± *SD*)	62.0 ± 8.7	64.0 ± 7.3	63.5 ± 8.1
Gender			
Female	12 (25.0)	5 (10.4)	17 (17.7)
Male	36 (75.0)	43 (89.6)	79 (82.3)
Marital status			
Married	42 (87.5)	44 (91.7)	86 (89.6)
Single	6 (12.5)	4 (8.3)	10 (10.4)
Educational level			
Illiterate	6 (12.5)	5 (10.4)	11 (11.5)
Literate	2 (4.2)	1 (2.1)	3 (3.1)
Primary school	33 (68.8)	27 (56.3)	60 (62.5)
High school	4 (8.3)	9 (18.8)	13 (13.5)
University	3 (6.3)	6 (12.5)	9 (9.4)
Residence			
City	19 (39.6)	16 (33.3)	35 (36.5)
County	23 (47.9)	24 (50.0)	47 (49.0)
Village	6 (12.5)	8 (16.7)	14 (14.6)
Living situation			
Alone	6 (12.5)	2 (4.2)	8 (8.3)
Spouse	19 (39.6)	26 (54.2)	45 (46.9)
Spouse and children	20 (41.7)	18 (37.5)	38 (39.6)
Relatives	2 (4.2)	2 (4.2)	4 (4.2)
Parents	1 (2.1)	—	1 (1.0)
Employment status			
Employed	14 (29.2)	5 (10.4)	19 (19.8)
Not employed	34 (70.8)	43 (89.6)	77 (80.2)
Chronic disease			
Yes	35 (72.9)	30 (62.5)	65 (67.7)
No	13 (27.1)	18 (37.5)	31 (32.3)
Diabetes ^a^			
Yes	21 (60.0)	19 (63.3)	40 (61.5)
No	14 (40.0)	11 (36.7)	25 (38.5)
Hypertension ^b^			
Yes	29 (82.9)	18 (60.0)	47 (72.3)
No	6 (17.1)	12 (40.0)	18 (27.7)
Chronic obstructive pulmonary disease ^b^			
Yes	—	4 (13.3)	4 (6.2)
No	35 (100.0)	26 (86.7)	61 (93.8)
Previous surgery			
Yes	28 (58.3)	24 (50.0)	52 (54.2)
No	20 (41.7)	24 (50.0)	44 (45.8)
Previous hospitalization			
Yes	40 (83.3)	35 (72.9)	75 (78.1)
No	8 (16.7)	13 (27.1)	21 (21.9)
Preoperative training			
Yes	24 (50.0)	27 (56.3)	51 (53.1)
No	24 (50.0)	21 (43.8)	45 (46.9)
Time of training ^a^			
Preoperative	24 (100.0)	27 (100.0)	51 (100.0)
Trainer ^a^			
Nurse	3 (12.5)	2 (7.7)	5 (9.8)
Physician	10 (41.7)	10 (37.0)	20 (39.2)
Nurse and physician	11 (45.8)	15 (55.6)	26 (51.0)
Adequacy of training ^a^			
Yes	18 (75.0)	19 (70.4)	37 (72.5)
Partially	6 (25.0)	8 (29.6)	14 (27.5)

^a^ Percentage of those with formal education was calculated (total *n* = 51).

^b^ Percentage of those with chronic diseases was calculated (*n* = 35, 30).

No between-group difference was identified in terms of preoperative anxiety levels regarding postoperative complications (*p* > .05; Table 2). However, a significant between-group difference was identified in terms of preoperative fear levels regarding postoperative complications, with patients in the referral group who did not experience gastrointestinal-related complications and those with lung-related complications, respectively, reporting significantly higher levels of fear (*p* < .05; Table 3).

**Table 2:** Between-Group Comparison of Preoperative Anxiety Levels by Postoperative Complication (*N* = 96)

Complication	Referral Group (*n* = 48)		Nonreferral Group (*n* = 48)		*p* ^a^
	*n* (%)	Anxiety Score Medians (IQR)	*n* (%)	Anxiety Score Medians (IQR)	
Complication development					
Yes	19 (39.6)	4 (2.0–12.0)	20 (41.7)	3 (1.3–10.5)	.30
No	29 (60.4)	7 (4.0–9.0)	28 (58.3)	5.5 (2.0–10.0)	.29
Bleeding					
Yes	5 (26.3)	4 (2.5–9.0)	7 (35.0)	3 (1.0–5.0)	.34
No	14 (73.7)	5 (2–12.25)	13 (65.0)	3 (1.0–17.0)	.48
Infection					
Yes	2 (10.5)	2 (6.0–0)	2 (10.0)	4 (3.0- 0)	.33
No	17 (89.5)	4 (2.0–10.0)	18 (90.0)	3 (1.0–12.0)	.48
Atrial fibrillation					
Yes	9 (47.4)	4 (2.0–11.0)	7 (5.0)	4 (1.0–22.0)	.60
No	10 (52.6)	4 (2.5–12.3)	13 (65.0)	3 (1.0–9.0)	.34
GIS-related					
Yes	—	—	1 (5.0)	—	NA
No	19 (100.0)	4 (2.0–12.0)	19 (95.0)	3 (1.0–6.0)	.19
Lung-related					
Yes	14 (73.7)	5 (2.0–12.3)	8 (40.0)	2.5 (0.5–4.5)	.09
No	5 (26.3)	4 (2.5–9.0)	12 (60.0)	3.5 (1.5–20.3)	.95
Cardiac-related					
Yes	4 (21.1)	5.5 (1.5–29.8)	4 (20.0)	2.5 (0.5–3.0)	.34
No	15 (78.9)	4 (2.0–12.0)	16 (80.0)	3.5 (1.3–14.3)	.74
Neurologic-related					
Yes	2 (10.5)	21.5 (6.0- 0)	2 (10.0)	4 (3.0- 0)	.33
No	17 (89.5)	4 (2.0–10.0)	18 (90.0)	3 (1.0–12.8)	.48
Renal-related					
Yes	1 (5.3)	—	—		NA
No	18 (94.7)	4 (2.0–9.0)	20 (100.0)	3 (1.3–10.5)	.44

*Note*. IQR = interquartile range (25–75); NA = nonapplicable; GIS = gastrointestinal.

^a^ Mann–Whitney *U* test.

**Table 3:** Between-Group Comparison of Preoperative Fear Levels by Postoperative Complication (*N* = 96)

Complication	Referral Group (*n* = 48)		Nonreferral Group (*n* = 48)		*p* ^a^
	*n* (%)	Fear Level Score Medians (IQR)	*n* (%)	Fear Level Score Medians (IQR)	
Complication development					
Yes	19 (39.6)	22 (14.0–36.0)	20 (41.7)	8 (0.5–23.3)	.06
No	29 (60.4)	27 (5.0–39.5)	28 (58.3)	20 (5.0–33.5)	.43
Bleeding					
Yes	5 (26.3)	12 (0–21.5)	7 (35.0)	8 (2.0–19.0)	1.000
No	14 (73.7)	25.5 (20.8–39.5)	13 (65.0)	16 (0–30.0)	.61
Infection					
Yes	2 (10.5)	55 (50.0–0)	2 (10.0)	28 (21.0–0)	.33
No	17 (89.5)	22 (13.0–26.5)	18 (90.0)	8 (0–20.3)	.07
Atrial fibrillation					
Yes	9 (47.4)	25 (16.5–43.0)	7 (5.0)	19 (5.0–48.0)	.60
No	10 (52.6)	22 (9.0–26.3)	13 (65.0)	8 (0–19.5)	.77
Gastrointestinal-related					
Yes	—	—	1 (5.0)	—	NA
No	19 (100.0)	22 (14.0–36.0)	19 (95.0)	8 (0–21.0)	**.03**
Lung-related					
Yes	14 (73.7)	24.5 (19.8–39.5)	8 (40.0)	12 (0.50–22.5)	**.02**
No	5 (26.3)	12 (0–29.0)	12 (60.0)	8 (1.0–32.3)	.87
Cardiac-related					
Yes	4 (21.1)	23 (22.0–51.0)	4 (20.0)	10 (0.5–22.5)	.11
No	15 (78.9)	21 (12.0–36.0)	16 (80.0)	8 (1.0–31.5)	.24
Neurologic-related					
Yes	2 (10.5)	55 (50.0–0)	2 (10.0)	17.5 (0–0)	.33
No	17 (89.5)	22 (13.0–26.5)	18 (90.0)	8 (1.5–21.8)	.11
Renal-related					
Yes	1 (5.3)	—	—	—	NA
No	18 (94.7)	22 (13.5–29.3)	20 (100.0)	8 (0.50–23.3)	.09

*Note*. IQR = interquartile range (25–75); NA = nonapplicable; Bold values are statistically significance (*p* < .05).

^a^ Mann–Whitney *U* test.

No statistically significant between-group differences were noted in postoperative pain levels on days 0, 1, and 2, or in the medians of preoperative anxiety and surgical fear scores (*p* > .05; Table 4).

**Table 4:** Between-Group Comparison of Postoperative Pain, Preoperative Anxiety, and Surgical Fear Levels (*N* = 96)

Scale	Referral Group (*n* = 48)	Nonreferral Group (*n* = 48)	*p* ^a^
	Median (IQR)	Median (IQR)	
Visual Analog Scale			
Postop 0	2 (2–4)	2 (2–4)	.960
Postop 1	3 (2–4)	3 (2–4)	.900
Postop 2	2 (2–3)	2 (2–4)	.930
Anxiety total	6 (4–9)	4 (2–10)	.132
Surgical Fear Scale total	24.5 (10.0–36.8)	16.5 (3.3–31.3)	.098
Long-term fear	10.0 (2.0–22.5)	5.0 (0–13.5)	.067
Short-term fear	10.5 (5.5–17.8)	9.5 (0–18.0)	.426

*Note*. IQR = interquartile range (25–75).

^a^ Mann–Whitney *U* test.

In the referral group, a significantly positive relationship was identified between anxiety and surgical fear (*p* < .05). Similarly, in the nonreferral group, a significantly positive relationship was identified between anxiety and surgical fear and a significantly negative relationship was identified between discharge time and anxiety (*p* < .05). Furthermore, for the referral group, a significant positive relationship was identified between surgical fear and pain (*p*= .05), and a significantly negative relationship was identified between discharge time and anxiety (*p* = .05; Table 5).

**Table 5:** Between-Group Comparison of the Relationship Between Preoperative Anxiety and Fear and, Respectively, Postoperative Pain and Selected Study Variables (*N* = 96)

Selected Variable and Scale	Referral Group (*n* = 48)				Nonreferral Group (*n* = 48)			
	Surgical Fear		Anxiety		Surgical Fear		Anxiety	
	*r*	*p*	*r*	*p*	*r*	*p*	*r*	*p*
Anxiety	.52	**<.001**			.39	**.005**		
Pain	.28	**.05**	.21	.156	−.16	.264	.03	.843
Length of stay in intensive care unit (days)	.20	.16	−.25	.87	.25	.08	.71	.63
Length of intubation (hours)	−.17	.90	−.10	.47	−.27	.85	−.44	.76
Length of mobilization (days)	−.25	.86	−.13	.93	.26	.85	.45	.75
Length of hospitalization (days)	.31	.83	−.27	**.05**	−.16	.26	−.42	**.003**

*Note*. Boldface data means *p* ≤ .05.

## Discussion

Anxiety, characterized by feelings of restlessness and tension arising from the anticipation of potential danger, typically initiates upon the disclosure of the need for surgical intervention and tends to intensify during hospitalization. In one prior study, high anxiety levels in patients were shown to positively relate to the way they were admitted for emergency surgery (Berhe et al., 2022). Although no significant difference in preoperative anxiety level was found between the two groups in this study, the referral group reported higher mean anxiety and fear scores than the nonreferral group.

Prior research into the relationship between preoperative anxiety and postoperative pain has yielded mixed findings (Kashif et al., 2022; Tadesse et al., 2022), with some, including one conducted in Turkey, finding no significant relationship (Yaman & Aygin, 2022), while others finding a significant relationship (Arslan Isik & Emir, 2022). Notably, the results of this study deviate from prior findings in observing no significant relationship between preoperative anxiety and postoperative pain in either patient group.

In this study, a significantly positive correlation was detected between preoperative surgical fear and postoperative pain levels among the patients referred to our hospital from outpatient clinics (the referral group). This finding is consistent with Arslan Isik and Emir (2022), who reported a significantly positive correlation between preoperative surgical fear and postoperative pain levels. However, no distinction was made in their study between referral and nonreferral patients. The uniqueness of this study comes from the categorization of patients based on admission source (i.e., outpatient clinics and our hospital). Therefore, unlike in this study, no statistically significant correlation was found between preoperative surgery fear level and postoperative pain in that previous work.

In Hernández-Palazón et al. (2018), no significant relationship was found between prolonged length of stay in the intensive care unit or hospital and high preoperative anxiety levels. In this study, no relationship was found between preoperative anxiety levels and complications such as postoperative neurological dysfunction and acute kidney injury. While Rodrigues et al. (2020) found no significant relationship between preoperative anxiety and postoperative complications, Gao et al. (2021) identified a significantly lower incidence of postoperative complications in patients with mild anxiety compared to those with no anxiety or moderate-to-severe anxiety. In this study, similar to those of Hernandez-Palazon et al. and Rodrigues et al., no significant relationship was found between preoperative anxiety and postoperative complications. Findings regarding the relationship between preoperative anxiety levels and postoperative complications vary in the literature. Notably, in this study, referral group participants with pulmonary-related complications and no preoperative gastrointestinal-related complications had a significantly higher mean level of surgical fear than their other participant peers. Also unique to this study, a significant relationship was identified between preoperative anxiety level and discharge time in both groups, potentially explainable by our exclusive focus on patients undergoing coronary artery bypass graft surgery.

In this study, referral group participants with pulmonary complications were found to have significantly higher levels of fear than their group peers. Surgical stress is known to cause changes in respiratory physiology, which affects lung volumes, respiratory drive, and muscle function and may increase the risk of postoperative pulmonary complications. Although surgical fear may not be the specific source of stress, surgical stress has been reported to trigger lung-related complications (Borsook et al., 2010; Sameed et al., 2021).

In this study, the effect of preoperative anxiety and fear levels on postoperative pain and complications between two groups of patients, that is, those referred to our hospital from outpatient clinics and those diagnosed and admitted by our hospital, scheduled for coronary artery bypass graft surgery, was investigated. The results identified no difference between the two groups in terms of pain, anxiety, or surgical fear. However, a significant correlation was found between anxiety and surgical fear scale scores within both groups. In addition, a significant correlation was found between discharge time and anxiety in the nonreferral group and significant relationships between the surgical fear scale score and, respectively, pain levels, discharge time, and anxiety scores were found in the referral group. Measuring the anxiety and fear levels of patients during the preoperative period, developing nursing care accordingly, and planning appropriate interventions may help reduce the risk of complications in the postoperative period.

### Limitations

This study has several limitations. The sample was relatively small and was drawn from a single hospital in one geographical region of Turkey. Therefore, the results should not be generalized beyond this specific population.
